# Integrative Analysis of Metabolome and Transcriptome Revealed Lutein Metabolism Contributed to Yellow Flower Formation in *Prunus mume*

**DOI:** 10.3390/plants12183333

**Published:** 2023-09-21

**Authors:** Aiqin Ding, Fei Bao, Xi Yuan, Jia Wang, Tangren Cheng, Qixiang Zhang

**Affiliations:** 1 Beijing Key Laboratory of Ornamental Plants Germplasm Innovation and Molecular Breeding, National Engineering Research Center for Floriculture, School of Landscape Architecture, Beijing Forestry University, Beijing 100083, China; 2 Beijing Laboratory of Urban and Rural Ecological Environment, Engineering Research Center of Landscape Environment of Ministry of Education, School of Landscape Architecture, Beijing Forestry University, Beijing 100083, China; 3 Key Laboratory of Genetics and Breeding in Forest Trees and Ornamental Plants of Ministry of Education, School of Landscape Architecture, Beijing Forestry University, Beijing 100083, China

**Keywords:** *Prunus mume*, yellow flower, transcriptome, metabolome, lutein, carotenoid biosynthesis, carotenoid cleavage dioxygenase 4

## Abstract

*Prunus mume* is a famous ornamental woody tree with colorful flowers. *P. mume* with yellow flowers is one of the most precious varieties. Regretfully, metabolites and regulatory mechanisms of yellow flowers in *P. mume* are still unclear. This hinders innovation of flower color breeding in *P. mume*. To elucidate the metabolic components and molecular mechanisms of yellow flowers, we analyzed transcriptome and metabolome between ‘HJH’ with yellow flowers and ‘ZLE’ with white flowers. Comparing the metabolome of the two varieties, we determined that carotenoids made contributions to the yellow flowers rather than flavonoids. Lutein was the key differential metabolite to cause yellow coloration of ‘HJH’. Transcriptome analysis revealed significant differences in the expression of carotenoid cleavage dioxygenase (*CCD*) between the two varieties. Specifically, the expression level of *PmCCD4* was higher in ‘ZLE’ than that in ‘HJH’. Moreover, we identified six major transcription factors that probably regulated *PmCCD4* to affect lutein accumulation. We speculated that carotenoid cleavage genes might be closely related to the yellow flower phenotype in *P. mume*. Further, the coding sequence of *PmCCD4* has been cloned from the ‘HJH’ petals, and bioinformatics analysis revealed that PmCCD4 possessed conserved histidine residues, ensuring its enzymatic activity. PmCCD4 was closely related to PpCCD4, with a homology of 98.16%. Instantaneous transformation analysis in petal protoplasts of *P. mume* revealed PmCCD4 localization in the plastid. The overexpression of *PmCCD4* significantly reduced the carotenoid content in tobacco plants, especially the lutein content, indicating that lutein might be the primary substrate for PmCCD4. We speculated that *PmCCD4* might be involved in the cleavage of lutein in plastids, thereby affecting the formation of yellow flowers in *P. mume*. This work could establish a material and molecular basis of molecular breeding in *P. mume* for improving the flower color.

## 1. Introduction

Flower color is a capital feature that attracts insects and birds for pollination and reproduction and protects flower organs from damage. Meanwhile, flower color extracts are widely applied in cosmetics, medical treatment, foods and other fields [1,2,3]. For ornamental plants, flower color is an essential determinant of quality, affecting ornamental and commercial value. Flower color is mainly defined by four pigments: flavonoids, carotenoids, betalains and chlorophyll [4,5]. The types, concentrations and composition of four pigments directly affect the flower color diversity. Among them, betalains only exist in *Caryophyllales* [6]. In nature, the petals of many plants contain chlorophyll at the early stage of development and appear green. The chlorophyll content gradually decreases until there is only a small amount of chlorophyll in the petals with flower development [7,8]. Flavonoids are widely found in plant flowers, conferring plants light yellow to blue-purple [9]. The flavonoids biosynthesis pathway has been intensively characterized, which is mainly controlled by structure genes and transcription factors, especially the MBW (MYB, bHLH and WD40-repeat) complex [10]. Carotenoids are natural pigments divided into carotene and xanthophyll, which belong to terpenoids [11]. Carotenoids contribute to flower pigmentation and adjust red, yellow and orange hues [12]. Meanwhile, it is essential in plant photosynthesis, plant hormone abscisic acid and strigolactone biosynthesis [13,14,15]. Therefore, exploring the composition and formation mechanism of flower colors is significant for germplasm innovation and molecular breeding of ornamental plants.

The stable accumulation of carotenoids is a complex process involving multiple genes. At present, the carotenoid biosynthesis pathway has been established, and most of the pathway genes have been identified in plants. The synthesis of carotenoids began with the direct precursor substance geranylgeranyl pyrophosphate (*GGPP*). Phytoene synthase (*PSY*) was the first key rate-limiting enzyme in the carotenoid biosynthesis pathway, determining the total amount of carotenoid metabolism. In rice, tobacco and tomato, *PSY* gene overexpression increased the carotenoid content and secondary metabolites of transgenic lines [16,17,18]. A critical branch of the carotenoid metabolism pathway was the cyclization of lycopene by lycopene ε-cyclase (*LCYE*) and lycopene β-cyclase (*LCYB*) [19]. The expression level and ratio of *LCYE* and *LCYB* are essential factors determining the branching direction of lycopene. In *Arabidopsis*, the *LCYE* function deficient in the lutein-deficient 2 (*lut2*) mutant caused the content of zeaxanthin, anther xanthin, and purple xanthin significantly increased in the β–branch [20]. Similarly, by using RNAi technology to inhibit the expression of the *LCYE* gene in sweet potato (*Ipomoea batatas*), the content of carotenoids, including β-cryptoxanthin, β-carotene and zeaxanthin, significantly increased [21]. However, there is no report on whether carotenoid synthesis genes are involved in the formation of flower color in *Prunus mume*.

Carotenoids accumulation is also affected by carotenoid degradative enzymes. Carotenoid cleavage dioxygenases (*CCDs*) are the crucial enzyme to degrade carotenoids, including *CCD* and 9-cis-epoxycarotenoid dioxygenase (*NCED*). CCD subfamily members are involved in the synthesis of natural active compounds, such as aroma volatiles and strigolactones, according to their substrate and cleavage position selectivity [22]. At present, *CCDs* have been identified with verified function from a large number of plant varieties, for example, *Arabidopsis* [23], tomato [24], *Vigna unguiculata* [25], *Petunia hybrida* [26,27], *Chrysanthemum* [28], *Brassica*, *Lycium chinense* [29,30], *Citrus* [31], and *Prunus persica* [32]. Among them, *CCD1* and *CCD4* were involved in the cleavage of carotenoids, providing unique colors, flavors and aromas for fruits and flowers. Moreover, a few transcription factors were found to directly regulate the expression of *CCDs*. In *Osmanthus*, *OfWRKY3*, *OfERF61* and *OfERF2* could bind *CCD1 or CCD4* promoters to regulate gene expression, thereby affecting the activities of carotenoid cleavage [33,34,35]. Moreover, the transcription factor complex CmAP3-CmPI-CmUIF1 could directly regulate *CmCCD4a-2* to modulate carotenoid metabolism in *chrysanthemum* [36].

*Prunus mume* Sieb. et Zucc is a woody plant belonging to the *Prunus* genus of the Rosaceae family. It is a famous traditional flower with colorful flowers, varied flower patterns, delicate fragrances and graceful postures in China. The flower color, including red, pink, white, yellow and multicolor, is a crucial character that determines the ornamental value of *P. mume*. Research on flower color started relatively late in *P. mume* and mainly focused on the anthocyanin pathway [37,38,39,40,41]. *P. mume* with a yellow flower is one of the most precious varieties. Meanwhile, the flowers are light yellow, and the yellow gradually fades as the flowers open. Therefore, breeding bright and stable yellow flowers has become one of the main breeding objectives in *P. mume.* However, specific substances and regulatory mechanisms of yellow flowers in *P. mume* are still unclear. Here, we combined transcriptome and metabolome to elucidate the metabolic components and molecular formation mechanism of the yellow flower. In addition, this work identified a color-formation-related gene, *PmCCD4*, which had the ability to degrade carotenoids. These findings could provide the scientific basis for the innovation of flower color breeding in *P. mume*.

## 2. Results

### 2.1. Description of Flower Color Parameters for the Yellow Variety

**Figure 1:**
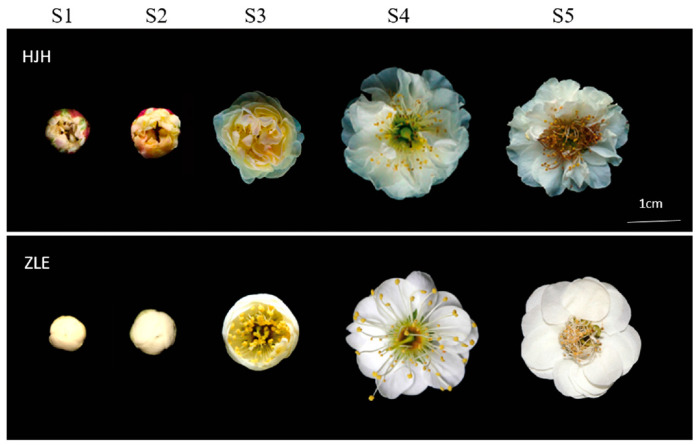
Phenotypes of *P. mume* ‘HJH’ and ‘ZLE’ flowers. ‘HJH’ means *P. mume* ‘Huangjinhe’ and ‘ZLE’ means *P. mume* ‘Zaolve’; S1, S2, S3, S4 and S5 represent small bud stage, big bud stage, half-flowering stage, whole-blooming stage and terminal florescence, respectively. The bar means 1 cm at the bottom-right corner.

The flower color of ‘HJH’ presents yellow, which is exceedingly rare in *P. mume.* Interestingly, the ‘HJH’ flower color showed dynamic changes as the petals bloomed (Figure 1). Following RHSCC estimate system (Table 1), petals at bud stage, S1 and S2 were yellow (5D), then the yellow color gradually faded. The middle part of flower was yellow, and periphery became white (2D) in the S3 stage. Finally, in the S4 and S5 stages, flowers turned white (155D, 155B). The chrominance of flower was described with the CIELAB system. As flower bloomed, the b*, C* and h values gradually decreased and rose slightly at S5. However, the trend in L* values was contrary to that in b* values. And the a* values remained unchanged. Compared with ‘HJH’, the flower color values of petals in the control variety ‘ZLE’ were more stable. Based on the color characteristics of ‘HJH’ petals, three stages with obvious color differences compared with check variety ‘ZLE’, S2, S3 and S4 were selected for the following research (Figure 1 and Table 1).

### 2.2. Identification of Flavonoids and Carotenoids in Yellow Flowers

**Figure 2:**
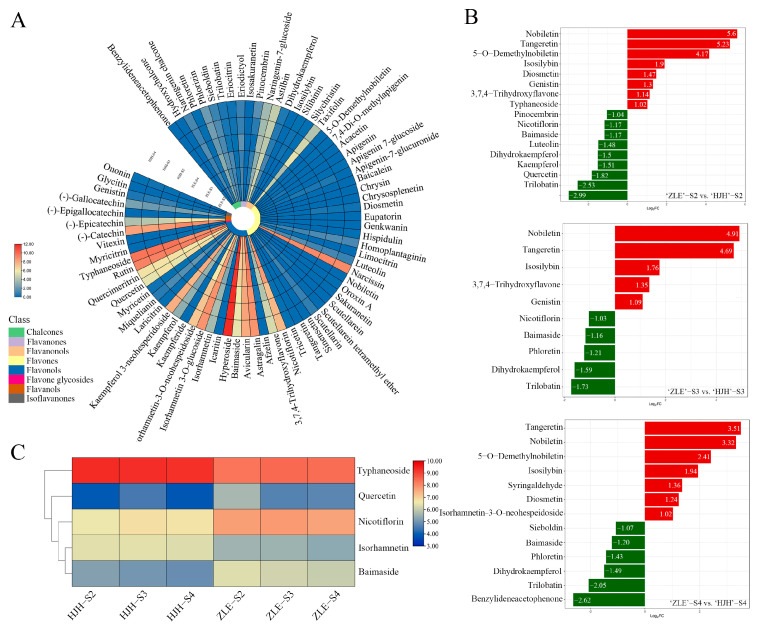
Differential flavonoid metabolites analysis between ‘HJH’ and ‘ZLE’ flowers. (**A**) Heatmap of the types and levels of flavonoids in two varieties at different stages. The colorful scales represent different classes. (**B**) Differential flavonoid metabolites in 3 comparison groups. (**C**) Heatmap of the main differential flavonoids in ‘HJH’ and ‘ZLE’ (contents > 10 μg/g DW). The colorful heatmap cells refer to the content of metabolites. Red to blue represents high to low content.

To explore the metabolites of the yellow flower coloration of ‘HJH’ and ‘ZLE’, the composition, concentration and accumulation patterns of flavonoids and carotenoids were analyzed with UPLC-MRM-MS/MS. Three bloom stages of two varieties were identified, including 74 flavonoids, which were classed into 7 chalcones, 4 flavanones, 7 flavanonols, 27 flavones, 21 flavonols, 1 flavone glycosides, 4 flavanols and 3 isoflavanones. The content of the flavonols was the most abundant, and the main flavonols were astragalin, hyperoside, rutin, etc., with hyperoside having the highest content (Supplementary Table S1 and Figure 2A). Next, we analyzed differential flavonoids in ‘HJH’ and ‘ZLE’. There were 17, 10 and 13 differential flavonoids discovered in ‘ZLE’-S2 vs. ‘HJH’-S2, ‘ZLE’-S3 vs. ‘HJH’-S3 and ‘ZLE’-S4 vs. ‘HJH’-S4, respectively, in which nobiletin, tangeretin, isosilybin, baimaside, dihydrokaempferol and trilobatin were detected in all of the three comparison groups (Figure 2B). A total of 20 differential flavonoid metabolites were detected, including 4 chalcones, 1 flavanone, 2 flavanonols, 6 flavones, 6 flavonols and 1 isoflavanones (Supplementary Table S2). The major differential metabolites were typhaneoside, isorhamnetin-3-O-neohespeidoside, nicotiflorin, quercetin and baimaside (contents > 10 μg/g DW). The contents of typhaneoside and isorhamnetin-3-O-neohespeidoside were higher in ‘HJH’, while that of quercetin, nicotiflorin and baimaside were lower in it. Moreover, the content of typhaneoside was the highest among all the flavonoids in the two varieties (Figure 2C).

**Figure 3:**
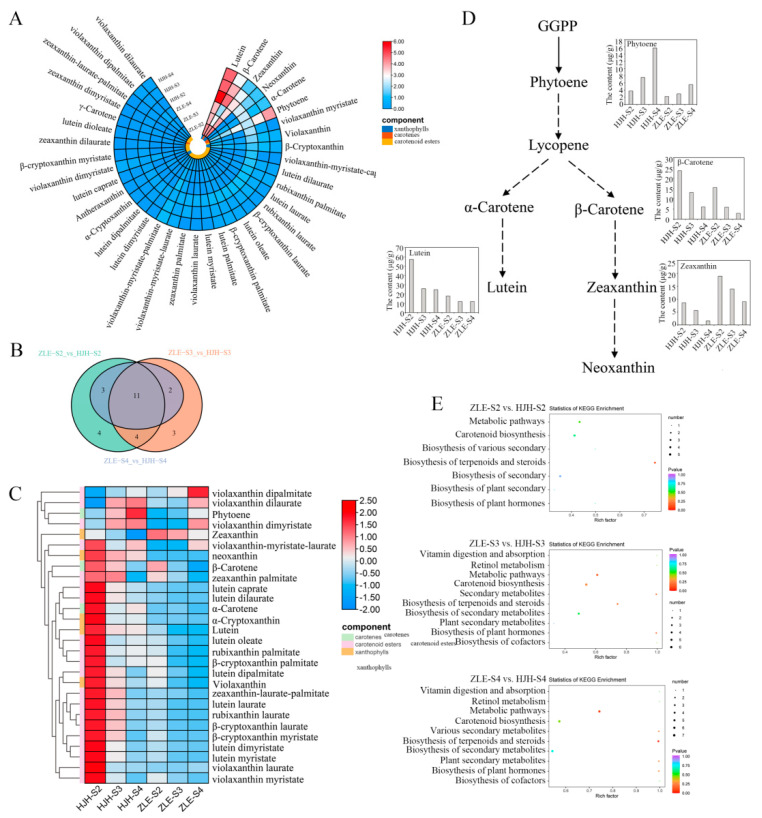
Differential carotenoids metabolites analysis of *P. mume* ‘HJH’ and ‘ZLE’ flowers. (**A**) Heatmap of the types and levels of carotenoids in two varieties at different stages. (**B**) Venn diagram of carotenoid metabolites. (**C**) Heatmap of differential carotenoid metabolites contents. The colorful scales represent different classes. The colorful heatmap cells mean content of metabolites. Red to blue represent high to low content. (**D**) The contents of major differential carotenoids (contents > 10 μg/g DW). (**E**) KEGG enrichment analysis of differential carotenoid metabolites.

A total of 37 carotenoid metabolites were detected, which can be divided into three categories: xanthophylls, carotenes and carotenoid esters (Figure 3A and Supplementary Table S3). The metabolites belonging to carotenoid esters were the most abundant, with up to 26, while the contents of xanthophylls were highest, which accounted for approximately 60%. The content of lutein was the highest in all stages. The total carotenoids were significantly accumulated at the S2 stage, with up to 124.23 μg/g (DW) in ‘HJH’. With the flowers opening, the content of total carotenoids decreased, especially in the S4 stage in both of the two varieties. Based on carotenoid metabolome data, 23, 21 and 17 differential carotenoids between ‘HJH’ and ‘ZLE’ were discovered in the S2, S3 and S4 groups, respectively. Twelve metabolites were found to exist in all three stages (Figure 3B). A total of 28 differential carotenoids existed in ‘HJH’ and ‘ZLE’, including 5 xanthophylls, 3 carotenes and 20 carotenoids esters (Supplementary Table S4 and Figure 3C). Among them, 26 differential carotenoids’ accumulation was higher in ‘HJH’. Based on the content of metabolites (>10 μg/g DW), four major differential carotenoids, namely lutein, β-carotene, zeaxanthin and (E/Z)-phytoene, were identified (Figure 3D). And the most abundant metabolite in the carotenoids was lutein. It decreased from 57.19 μg/g (DW) at the S2 stage to 24.62 μg/g (DW) at the S4 stage, which declined from 3.22 times to 2.06 times compared to ‘ZLE’. Among the differential carotenoid metabolites, lutein and β-carotene were the important carotenoids, and their contents accounted for 70.53% of the total carotenoids at the S2 stage. In addition, besides affecting flower color, the carotenoid metabolic pathway is closely related to the synthesis of some hormones and secondary metabolites. As shown in Figure 3E, carotenoid metabolites were significantly enriched in the plant hormones, terpenoids and steroids biosynthesis.

### 2.3. Lutein Determined the Yellow Flower Coloration in P. mume

**Figure 4:**
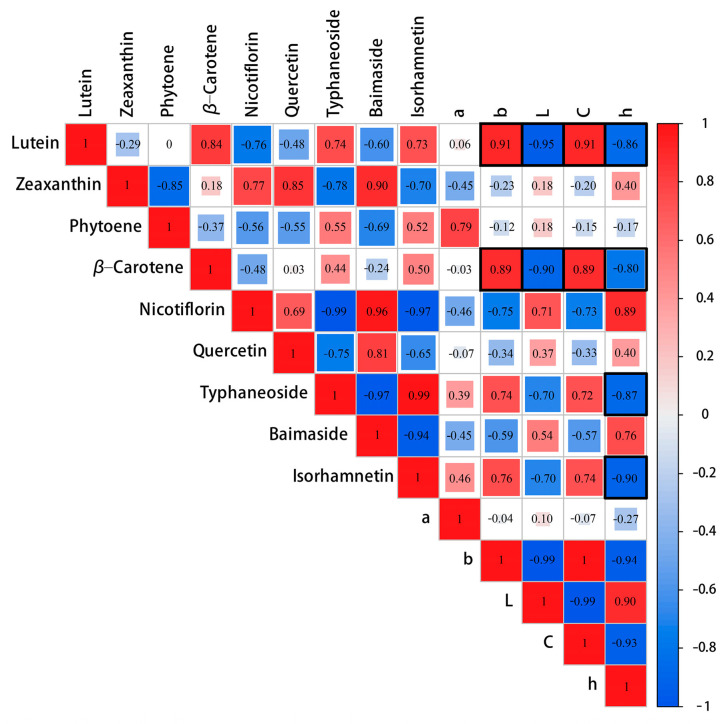
Correlation heatmap between color parameters and pigments at three flowering stages of ‘HJH’ and ‘ZLE’. Red indicates positive correlation and blue indicates negative correlation. The numbers within the rectangles represent Pearson correlation coefficients.

To study the contribution of different pigments to the coloration of yellow flowers, fourteen indicators that included the L*, a*, b*, C* and *h* value, five major differential flavonoids and four major differential carotenoids were selected. We analyzed the correlation among the fourteen indicators by calculating their Pearson correlation coefficients and visualized the results using a correlation heat map (Figure 4). The results indicate a highly significant negative correlation (*p* < 0.01) between the L* value and lutein, as well as a significant negative correlation (*p* < 0.05) with β-carotene. However, none of the five major differential flavonoids were found to have a significant correlation with the L* value. The b* and C* values were found to have a significant positive correlation with lutein and β-carotene (*p* < 0.05), while no significant correlation was observed between these values and the five major differential flavonoids. The *h* value exhibited a significant negative correlation (*p* < 0.05) with lutein, β-carotene, typhaneoside and isorhamnetin-3-O-neohespeidoside. There was no significant correlation observed between the a* value and the differential flavonoids or differential carotenoids. The correlation results indicated that the levels of carotenoids and flavonoids were both closely associated with the color of flower in *P. mume*. This suggests that these compounds play a significant role in determining the flower coloration of *P. mume*.

To further investigate the critical pigments that influenced the color formation of *P. mume*, multiple stepwise regression analysis was conducted using the L*, a*, b*, C* and *h* values as the response variables and nine differential compounds as predictors. The regression equations were as follows:

L* = 98.983 − 0.266 × Lutein (R2 = 0.886, *p* = 0.03)

b* = 0.461 × Lutein (R2 = 0.790, *p* = 0.011)

C* = 9.984 + 0.436 × Lutein (R2 = 0.785, *p* = 0.012)

According to the equation analysis, lutein was the principal pigment that influenced the L*, b* and C* values. Lutein had a significant negative effect on the color lightness (L*), meaning that the higher lutein content, the lower lightness of the flower color. The concentration of lutein in the petals significantly positively impacted b*. This means that as the concentration of lutein increases, the petals become more yellow in color. C* was positively correlated with lutein. Overall, lutein was a crucial factor in the yellow formation of *P. mume* and significantly affected the lightness and chroma of the petals.

### 2.4. Transcriptome Sequencing, Annotation and Analysis of DEGs

**Figure 5:**
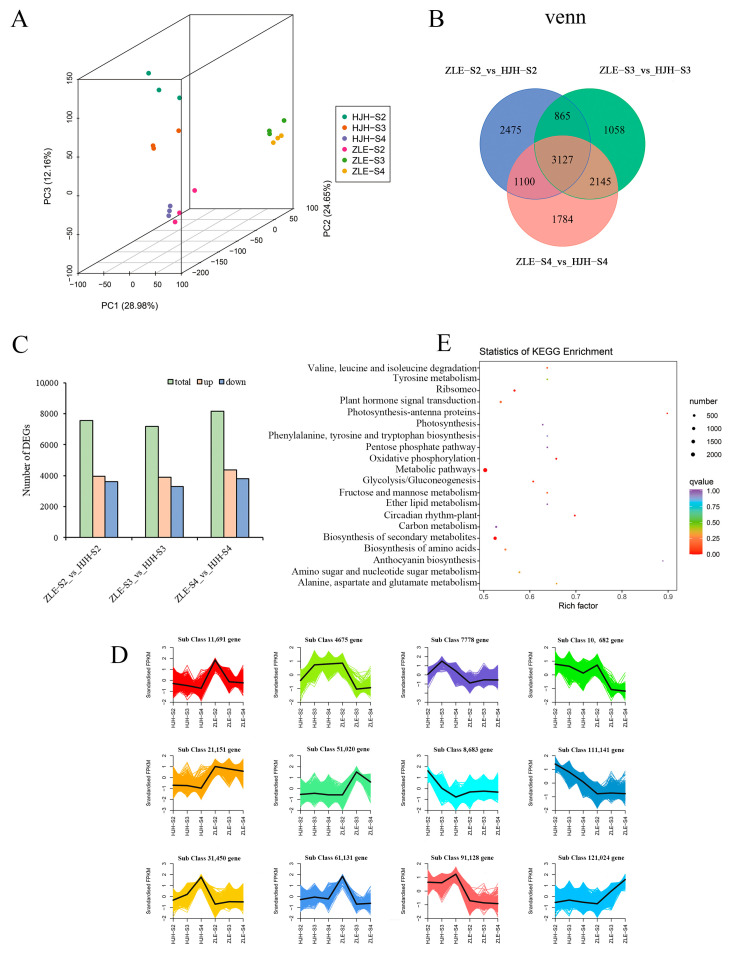
Transcriptome quality analysis of *P. mume* ‘HJH’ and ‘ZLE’ at S2, S3 and S4. (**A**) Principal component analysis of samples. (**B**) Venn diagrams of DEGs among different groups. (**C**) The number of DEGs. (**D**) Top 20 KEGG pathways enrichment analysis. (**E**) K-means cluster analysis of gene expression. The *x*-axis means samples, and the *y*-axis denotes gene expression of centralization and standardization. Different classes are expressed by colored lines. The samples with the same color represent three biological replications of the same period.

The transcriptome of 18 samples was sequenced and analyzed to explore gene expression between ‘HJH’ and ‘ZLE’. Through filtering, 7 G high-quality clean bases were obtained from raw reads in each sample. Then, we mapped clean reads to the reference genome of *P. mume*, and 81.97–93.97% were matched. The Q30 was more remarkable than 91.51%, and the GC content ranged from 45.2% to 46.23% (Supplementary Table S5). A total of 25, 419 genes were annotated with seven databases (GO, KEGG, KOG, NR, Pfam, SwissProt and Trembl), reaching an annotation rate of at least 73% (Supplementary Table S6). The correlation index R^2^ (Pearson’s correlation coefficient R) of biological repetition was greater than 0.89 (Supplementary Figure S1A). The PCA plot displayed that PC1 and PC2 contributed 53.63% and the two varieties were able to obviously separate (Figure 5A). The above results showed that the transcriptome data were reliable for subsequent analysis.

Based on |log2Fold Change| ≥ 1 and FDR < 0.05 (Supplementary Figure S2), 12,554 DEGs were screened in ‘ZLE’-S2 vs. ‘HJH’-S2, ‘ZLE’-S3 vs. ‘HJH’-S3 and ‘ZLE’-S4 vs. ‘HJH’-S4. The distribution of DEGs among the three groups was relatively balanced (Figure 5B). Additionally, there was a higher number of upregulated DEGs compared to downregulated DEGs (Figure 5C). According to K-means cluster analysis, DEGs were clustered into 12 categories (Figure 5D). Compared with ‘ZLE’ gene-expression levels in ‘HJH’ were higher in subclass 3, 7, 9 and 11. However, the trend in subclass 2, 5 and 12 was opposite. It is possible that these genes play important roles in the differences between the two varieties. All DEGs were enriched 57 GO terms through GO analysis system and extensively participated in cellular component, biological processes and molecular function (Supplementary Figure S1B). In the KEGG pathways, total DEGs were mapped to 140 pathways and the top 20 were significantly enriched in metabolic pathways and biosynthesis of secondary metabolites (Figure 5E). Three groups, ‘ZLE’-S2 vs. ‘HJH’-S2, ‘ZLE’-S3 vs. ‘HJH’-S3 and ‘ZLE’-S4 vs. ‘HJH’-S4, were significantly enriched in the top 20 KEGG pathways (*p* value ≤ 0.05) (Supplementary Figure S3). Among them, the carotenoid pathway (ko00906) was significantly enriched in the three comparison groups. Notably, we found 50 DEGs in the carotenoid pathway.

### 2.5. Structural Genes Associated with Carotenoid Biosynthesis

**Figure 6:**
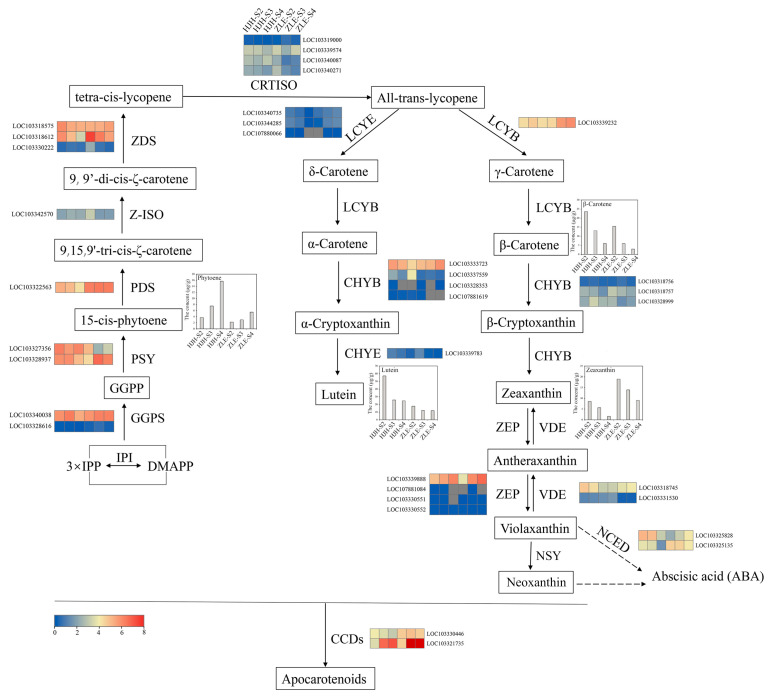
Structural genes of carotenoid biosynthesis pathway in *P. mume* ‘HJH’ and ‘ZLE’. GGPS, geranylgeranyl diphosphate synthase; *PSY*, phytoene synthase; *PDS*, phytoene desaturase; *ZDS*, ζ-carotene desaturase; *CRTISO*, carotenoid isomerase; *ZISO*, 15-cis-zeta-carotene isomerase; *LCYE*, lycopene epsilon cyclase; *LCYB*, lycopene beta cyclase; *CHYE*, carotene epsilon-monooxygenase; *CHYB*, beta-carotene hydroxylase; *ZEP*, zeaxanthin epoxidase; *VDE*, violaxanthin de-epoxidase; *NSY*, neoxanthin synthase; *CCD*, carotenoid cleavage dioxygenases; *NCED*, 9-cis-epoxycarotenoid dioxygenase. The colored scale represents the relative expression levels. Red means high expression, while blue means low expression.

Carotenoid accumulation led to the flower coloration difference between ‘HJH’ and ‘ZLE’. Thus, we constructed a pathway map based on all genes related to carotenoid biosynthesis and degradation (Figure 6). GGPP is a direct precursor of carotenoid biosynthesis, forming through the 2-C-methyl-D-erythritol-4-phosphate (MEP) pathway. GGPP is transformed into lycopene by PSY, phytoene desaturase (PDS), 15-cis-zeta-carotene isomerase (ZISO), ζ-carotene desaturase (ZDS) and carotenoid isomerase (CRTISO) [42]. To analyze the relationship between the content difference of carotenoids and genes in ‘HJH’ and ‘ZLE’, we compared the expression patterns with these genes. The results show that the expression level of *PSY* (LOC103327356) was higher in ‘HJH’ than that in ‘ZLE’. However, the expression profiles of *PSY* (LOC103328937), *PDS* (LOC103322563) and *ZDS* (LOC103318612) in ‘ZLE’ were higher than that in ‘HJH’.

Lycopene cyclase *LCYE* and *LCYB* are necessary carotenoid branching enzymes. Their expression levels and quantity ratio are important factors determining the direction of lycopene branching. *LCYE* or *LCYB* decide the category of δ-carotene or γ-carotene, respectively. Then, δ-carotene is converted to α-carotene by *LCYE*, and γ-carotene turns into β-carotene by *LCYB*. The expression of *LCYE* was no apparent difference between the two varieties. Strikingly, only one unigene that encoded *LCYB* (LOC103339232) was detected, while there were three unigenes that encoded *LCYE*. The ratio of the two genes promoted the notion that the ε-branch contained high lutein content, which was consistent with the metabolome. Comparing the expression patterns of *LCYB* in two varieties, we found that the expression level of *LCYB* in ‘ZLE’ was higher. *LCYB* might promote the synthesis of zeaxanthin downstream, resulting in a higher zeaxanthin content in ‘ZLE’ than ‘HJH’. In addition, there were no differences in the expression patterns of carotene epsilon-monooxygenase (*CHYE*), beta-carotene hydroxylase (*CHYB*), zeaxanthin epoxidase (*ZEP*) and violaxanthin de-epoxidase (*VDE*) between the ‘HJH’ and ‘ZLE’ varieties.

CCDs can cleavage carotenoids to different apocarotenoids, which are one of the main pathways for carotenoids degradation in organisms [43]. Two *CCD* genes were detected: *CCD1* (LOC103330446) and *CCD4* (LOC103321735). *CCD1* showed a correspondingly stable expression in various ‘HJH’ or ‘ZLE’ stages. Nevertheless, the expression of *CCD1* was upregulated in ‘ZLE’ compared to ‘HJH’. In contrast, the expression profile of *CCD4* increased with the flower blooming, in two varieties. Notably, the expression level of *CCD4* in ‘ZLE’ rapidly rose and was 22.5 times higher than that of ‘HJH’ at S3. This implied that *CCD1* and *CCD4* might be related to the flower coloration differences between the ‘HJH’ and ‘ZLE’ varieties at different stages. It was possible that the low expression of *CCD1* and *CCD4* in S2 and S3 of ‘HJH’ led to the accumulation of carotenoids in flowers, causing them to turn yellow.

*NCEDs* can degrade carotenoids and participate in the ABA pathway. We found two *NCED* genes in the transcriptome. *NCED3* (LOC103325828) and *NCED5* (LOC103325135) had a similar expression pattern in ‘HJH’. Furthermore, *NCED5* (LOC103325135) was expressed at a lower level in ‘HJH’ than ‘ZLE’.

**Figure 7:**
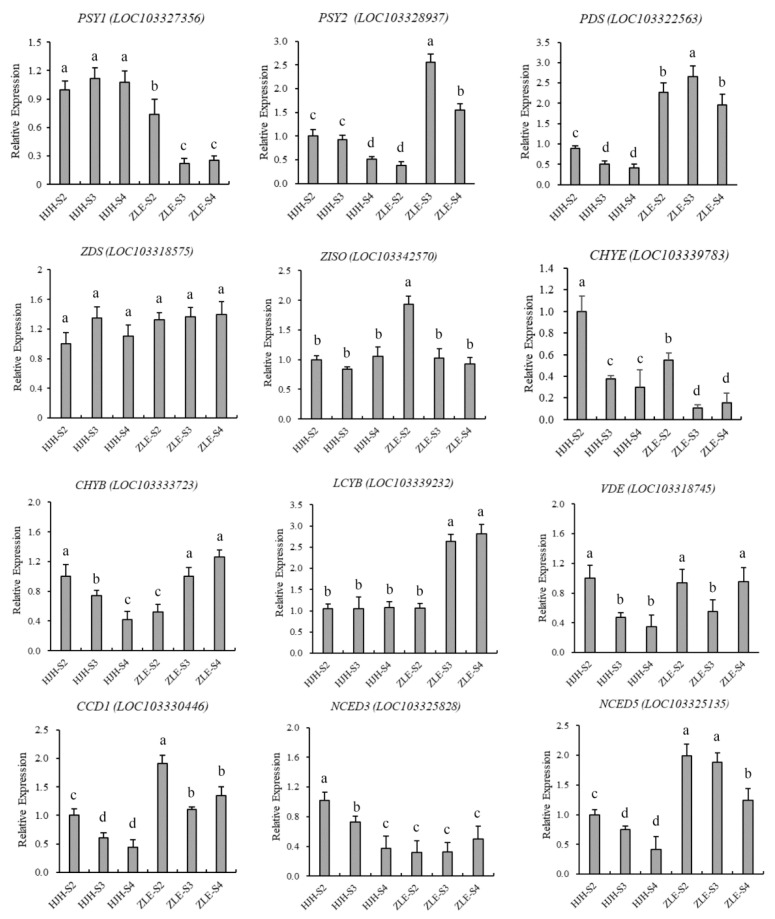
qRT-PCR analysis of selected candidate genes related to carotenoid biosynthesis pathway in *P. mume*. Standard deviation error bars represent three independent replicates. Statistically significant differences (*p* < 0.05) are denoted by different letters (a–d).

To further verify the above results, we randomly studied 12 genes involved in the carotenoid synthesis pathway for qRT-PCR analysis. The results show that gene-expression trends were consistent with the transcriptome data (Figure 7).

### 2.6. Potential Transcriptional Regulation Mechanisms

**Figure 8:**
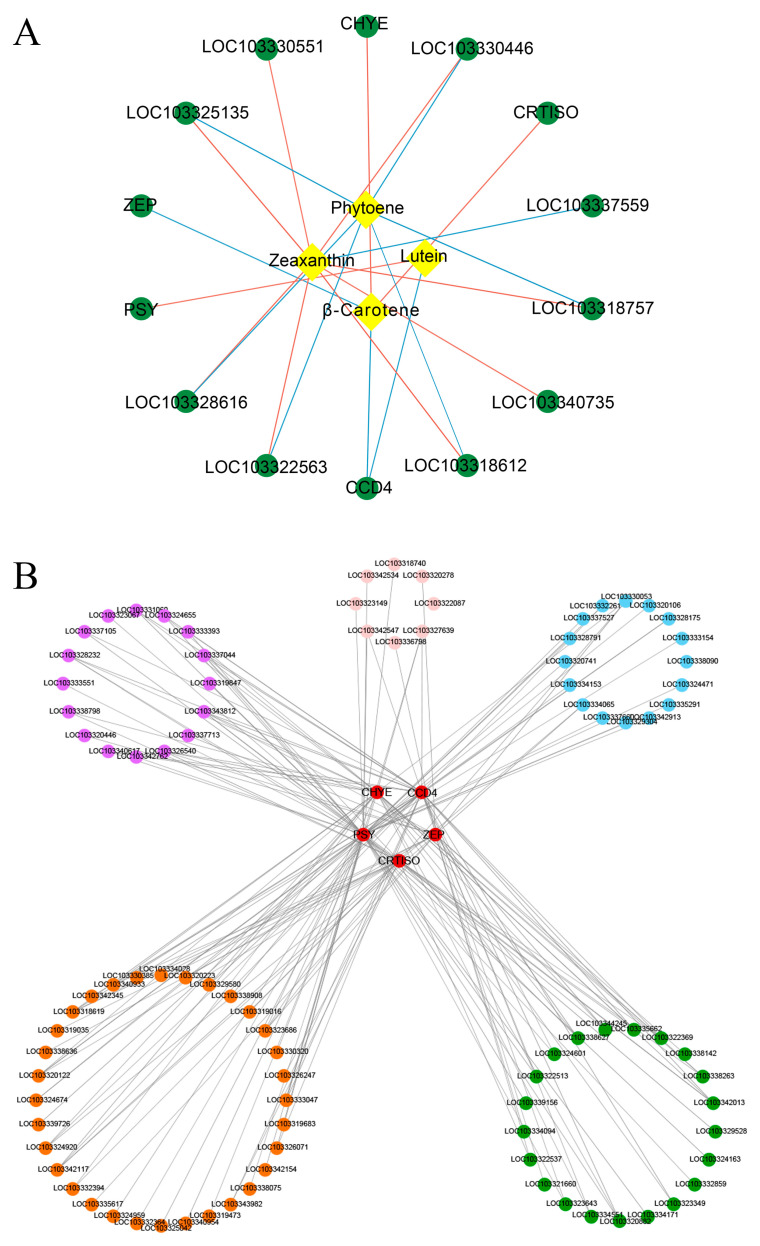
Construction of regulatory networks of carotenoid biosynthesis. (**A**) Establishment of co-expression network with structural genes and differential carotenoid metabolites. Green circles indicate structural genes and orange squares indicate differential carotenoid metabolites. Red lines denote positive correlation and blue lines denote negative correlation. Line thickness means the strength of correlation. (**B**) Co-expression analysis of screened genes and differential transcription factors (TFs). Red circles indicate screened genes, purple circles indicate ERF TFs, pink circles indicate MYB TFs, blue circles indicate WRKY TFs, green circles indicate bHLH TFs and orange circles indicate other TFs. Line thickness means the strength of correlation.

A network was drawn using selected 35 carotenoid pathway genes and 4 differential carotenoids (lutein, β-carotene, phytoene, and zeaxanthin) to clarify the correlation between genes and carotenoids in *P. mume* (Figure 8A). PCC (Pearson correlation coefficient) was used to estimate the relationships between genes and metabolites. The network showed that 14 genes significantly correlated with four different carotenoids. And 21 significant correlation pairs (|PCC|≥ 0.8 and *p*-value ≤ 0.05) were detected (Supplementary Table S8). Lutein is only correlated with two genes, while zeaxanthin is correlated with eight genes. There are five genes, namely *PSY* (LOC103327356), *CRTISO* (LOC103340271), *CHYE* (LOC103339783), *ZEP* (LOC103339888), and *CCD4* (LOC103321735), that are significantly related to the central differential carotenoid lutein and β-carotene. These genes likely play a crucial role in the biosynthesis or metabolism of these carotenoids. In particular, *CCD4* was correlated with both lutein and β-carotene. These results suggest that the accumulation of carotenoids in *P. mume* was regulated by multiple genes, with the *CCD4* gene potentially playing a primary role.

To further explore the regulatory mechanism of TFs in carotenoid biosynthesis, we constructed a network between carotenoid pathway genes and differentially expressed transcription factors (TFs). Five screened structural genes (*PSY*, *CRTISO*, *CHYE*, *ZEP* and *CCD4*) were used as seed nodes. Compared with the PlantTFDB database, 253 differentially expressed TFs were identified. Genes and transcription factors were used for correlation analysis. There were 93 TFs of 19 TF families that had a significant correlation with five genes. Among 19 TF families, bHLH (21 unigenes) was the most abundant, followed by ERF (16 unigenes), WRKY (16 unigenes) and MYB (8 unigenes) (Supplementary Table S9). Figure 8B shows that 170 significant correlation pairs (|PCC| ≥ 0.8 and *p*-value ≤ 0.05) were detected (Supplementary Table S10). Among 93 TFs, 33 were involved in the regulation of *CCD4*. *bHLH63* (LOC103335662), *ASIL1* (LOC103333047), *bHLH148* (LOC103323349), *MYB114* (LOC103327639), *RAP2-7* (LOC103324655) and *ERF3* (LOC103326540) were positively correlated with *CCD4* (PCC ≥ 0.9 and *p* value ≤ 0.05). Moreover, the connection strength between TFs and the target genes was also evaluated. The *bHLH63* (LOC103335662), which belongs to the bHLH family, had the strongest connection with *CCD4*.

### 2.7. Overexpression of PmCCD4 Decreased Carotenoids Content in Tobacco

**Figure 9:**
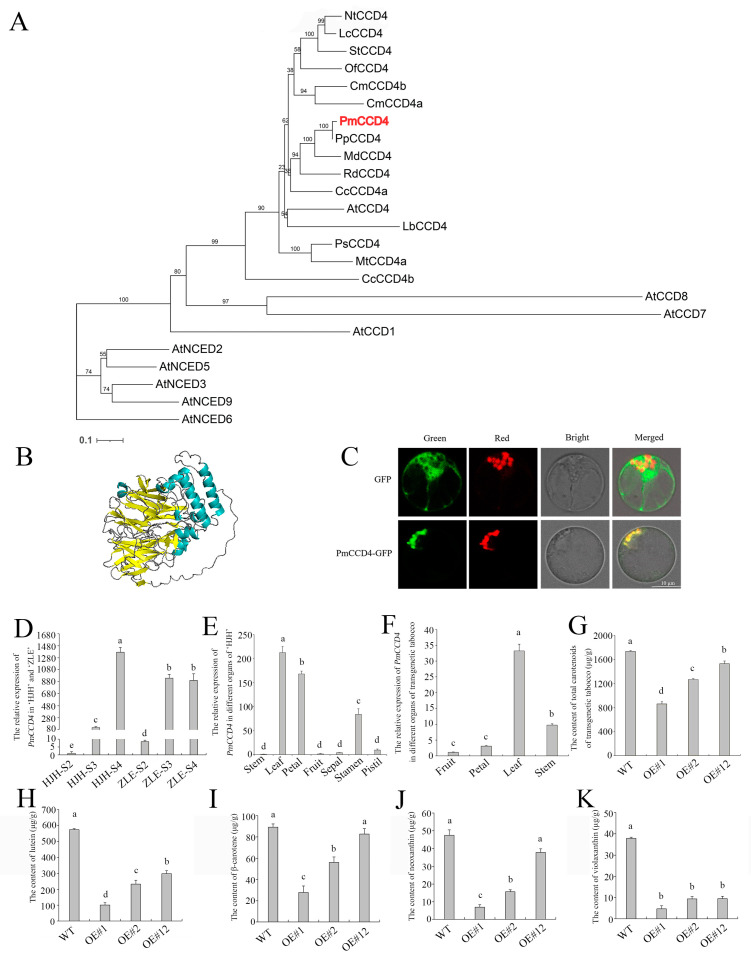
Analysis and function of PmCCD4. (**A**) Phylogenetic tree of CCD proteins from different plants. The GenBank accession numbers of the CCD proteins were as follows: *Arabidopsis thaliana* AtCCD1 (NP191911.1), AtCCD4 (NP193652.1), AtCCD7 (NP182026.5), AtCCD8 (NP195007.2), AtNCED2 (NP193569), AtNCED3 (NP_188062.1), AtNCED5 (NP_174302.1), AtNCED6 (NP_189064.1), AtNCED9 (NP_177960.1); *Citrus clemetina* CcCCD4a (DQ309330), CcCCD4b (DQ309331); *Chrysanthemum morifolium* CmCCD4a (ABY60885), CmCCD4b (BAF36656); *Lilium brownie* LbCCD4 (AB733097); *L. chinense* LcCCD4 (KM016897); *Malus domestica* MdCCD4 (ABY47995.1); *Medicago truncatula* MtCCD4a (XP003612460); *Nicotiana tabacum* NtCCD4 (AEI61930.1); *Osmanthus fragrans* OfCCD4 (ABY60887.1); *P. persica* PpCCD4 (AGL08676.1); *Pisum sativum* PsCCD4 (BAC10552); *Rosa damascena* RdCCD4 (ABY60886); *Solanum tuberosum* StCCD4 (XM_006359904.2); PmCCD4 is represented in red. (**B**) Predicted 3D structure of PmCCD4 protein. teal-helix represents α-helices; yellow-sheet represents β-sheet; gray line represents loop. (**C**) Subcellular localization of PmCCD4 protein. Green, GFP channel; Red, plastid; Bright, bright field image; Merged, merged image. (**D**) The expression level of *PmCCD4* in ‘HJH’ and ‘ZLE’. (**E**) The expression level of *PmCCD4* in different organs of ‘HJH’. (**F**) The expression level of *PmCCD4* in different organs of transgenic tobacco lines. (**G**) The total carotenoid content of three transgenic tobacco lines (OE-1, OE-2 and OE-12). (**H**–**K**) The contents of lutein, β-carotene, neoxanthin and violaxanthin of three transgenic tobacco lines (OE-1, OE-2 and OE-12). Standard deviation error bars represent three independent replicates. Statistically significant differences (*p* < 0.05) are denoted by different letters (a–e).

To verify the conjecture of *PmCCD4*, the 1794-bp open reading frame of *PmCCD4* corresponding to 587 amino acids was isolated from flowers at the anthesis stage. The phylogenetic analysis showed that PmCCD4 had the closest homologous relationship with PpCCD4. The amino acid sequence of PmCCD4 was 98.16% identified with PpCCD4 (Figure 9A). Sequence analysis indicated that PmCCD4 possessed a conserved RPE65 domain common to the CCD family. Simultaneously, PmCCD4 contained four highly conserved histidines and three glutamates or aspartates, required for CCD enzyme activity (Supplementary Figure S4).

Then, we used SWISS-MODEL and PyMol to build a spatial structure of the PmCCD4 protein. The model revealed that the PmCCD4 protein contained ten α-helices and multiple β-sheets (Figure 9B). The structure was the same as other known functional CCDs. The PCLR soft-predicted that PmCCD4 had chloroplast targeting, indicating that PmCCD4 may be located on the plastid to carry out its function. To determine the subcellular localization of the PmCCD4 protein, the vector pCAMBIA1300-*PmCCD4*-GFP was constructed and transferred into the petal protoplast of *P. mume*. Meanwhile, the *Arabidopsis* plastid localization gene PT-RK was transformed into protoplasts as a control. Figure 9C shows that the green fluorescence position of PmCCD4-GFP overlaps with the red fluorescence of PT-RK to form yellow fluorescence. The results reveal that PmCCD4 is located in the plastid of the petal cells.

According to the transcriptome analysis, there was a difference in the expression levels of *PmCCD4* in the ‘HJH’ and ‘ZLE’ varieties. The expression patterns of *PmCCD4* were verified on different flowering stages between two varieties. The analysis revealed that the expression level of *PmCCD4* differed significantly in both varieties. The expression level of *PmCCD4* increased gradually with the blooming of the flowers (Figure 9D). At the S2 and S3 stages, the expression level of *PmCCD4* in ‘ZLE’ was eight times higher than in ‘HJH’. However, at stage S4, the expression level of *PmCCD4* was higher in ‘HJH’ than ‘ZLE’, which correlated with the reduction in carotenoid content in ‘HJH’-S4. The expression levels of *PmCCD4* in different tissues of ‘HJH’ were found to vary significantly (Figure 9E). The expression levels of *PmCCD4* were higher in the petals and leaves, while they were lower in the stems and fruits. This suggests that *PmCCD4* may have tissue-specific functions or regulation. Additionally, the expression level of *PmCCD4* was 8.7 times higher in the stamen than in the pistil, speculating that it may play a role in another development or function.

Next, pCAMBIA1300-*PmCCD4* was overexpressed in tobacco using the leaf disk transformation method to test function. According to PCR amplification, the presence of the target gene was confirmed. The *PmCCD4* was expressed in the stems, leaves, petals and fruits of tobacco according to qRT-PCR experiments, indicating that it might exert function in different tissues. The expression level of *PmCCD4* was highest in leaves, reaching 33-fold compared to that in fruits (Figure 9F). The total carotenoid content in transgenic tobacco leaves was detected by UV spectrophotometry. Compared with wild-type tobacco, the total carotenoid content of transgenic tobacco significantly decreased, especially in OE-1, which decreased by two times (Figure 9G). Next, we employed the HPLC method to measure the content of various carotenoids in transgenic tobacco leaves. As shown in Figure 9H–K, the contents of lutein, β-carotene, violaxanthin and neoxanthin exhibited a reduction in *PmCCD4* overexpressing tobacco plants. Among them, the decrease in lutein content was the strongest. In comparison to WT, the overexpression of *PmCCD4* in tobacco resulted in a remarkable 5.8-fold decrease in lutein content. These findings suggest that PmCCD4 can degrade carotenoids in tobacco. And lutein might be the primary substrate for PmCCD4.

## 3. Discussion

**Figure 10:**
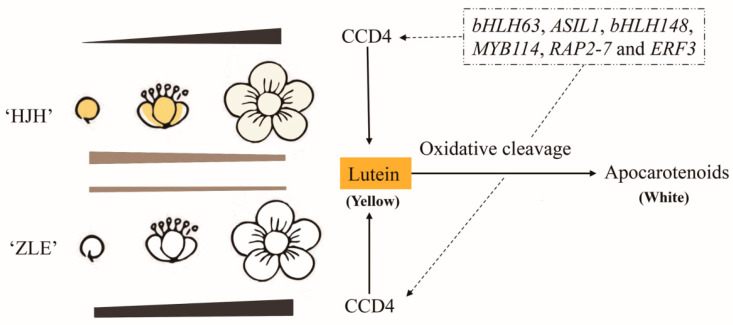
A hypothetical model for the formation of yellow flowers in *P. mume*.

*P. mume* is a famous ornamental tree, and its colorful flowers are a major character in determining ornamental value. Presently, research about flower color focuses on the anthocyanin pathway in *P. mume* [37,38,39,40,41]. However, few studies have been carried out on the formation mechanism of yellow flower in *P. mume*. The specific substances and regulation mechanism of yellow flowers are still unclear. In this study, we explored transcriptome and metabolome between ‘HJH’ with yellow flowers and ‘ZLE’ with white flowers. Comparing the metabolites, lutein was the key differential metabolites to cause yellow coloration of *P. mume*. Combined with transcriptome, we summarized a hypothetical model for the formation of yellow flowers in *P. mume* (Figure 10).

### 3.1. Impact of Flavonoids and Carotenoids on Yellow Flowers Coloration in P. mume

Flavonoids are the most well-studied plant pigments. Chalcone, flavones and flavonols mainly endow yellow flowers. *Dianthus caryophyllus*, *Cyclamen persicum*, *Catharanthus* and *Paeonia* yellow flowers are closely related to flavones [13,44,45,46]. In our research, 74 flavones were identified from *P. mume* ‘HJH’ and ‘ZLE’. Flavonols were the most abundant in two varieties. The result was similar with the pigment composition of yellow *Paeonia* [46]. Five major differential flavonoids metabolites were detected between ‘HJH’ and ‘ZLE’ (contents > 10 μg/g). Correlation analysis revealed that there was no significant correlation between L*, a*, b*, C* and the five differential flavonoids. But the h value exhibited a significant negative correlation (*p* < 0.05) with typhaneoside and isorhamnetin-3-O-neohespeidoside. We speculate that flavonoids might be an auxiliary pigment of yellow *P. mume*.

Carotenoids are a class of critical liposoluble pigments that exist widely in nature. Carotenoids confer flowers, fruits and other organs of advanced plants’ brilliant colors, attracting birds and insects to participate in plant pollination and seed dispersal [14]. For the study, 37 carotenoid metabolites were detected. And lutein was the highest at all stages. The variety and relative abundance of carotenoids determined flower color in plants [47,48,49,50,51]. Our carotenoid metabolome showed that 23, 21 and 17 differential carotenoids were discovered in the S2, S3 and S4 groups between ‘HJH’ and ‘ZLE’, respectively. A total of four major differential carotenoids existed in two varieties. Correlation analysis demonstrated that L*, b*, C* and *h* were significantly correlated with lutein. Further, multivariate stepwise regression analysis revealed that lutein was the vital pigment responsible for the yellow color of *P. mume*.

### 3.2. Carotenoid Biosynthesis in P. mume

To explore the accumulation pathway of carotenoids in *P. mume*, transcriptome sequencing and differential gene analysis were carried out. At present, there are few transcriptomes about *P. mume* with yellow flowers. In this study, three flowering stages of ‘HJH’ and ‘ZLE’ were detected by de novo sequencing. High-quality assembly data enriched the candidate gene pool for flower color improvement in *P. mume*.

Carotenoid biosynthesis is a complex process involving many enzymes. Carotenoids synthesis begins with the direct precursor GGPP. The GGPP is transformed into the first carotenoid phytoene by *PSY*. Then, the phytoene is converted to lycopene by *PDS*, *ZISO ZDS* and *CRTISO* [52]. An essential branch of the carotenoid metabolic pathway is the cyclization of lycopene by *LCYE* and *LCYB* [19,53,54,55]. Notably, three unigenes encoded *LCYE*, while only one unigene encoded *LCYB*. The ratio of the two genes promoted the notion that the ε-branch contained high lutein content, which was consistent with the metabolome. In this research, the expression level of carotenoid synthesis genes had no significant difference in the two varieties, which indicates that carotenoid synthesis genes were not the cause of the color difference.

However, *CCD* genes related to carotenoid degradation had significant expression in the two varieties. Significantly, the expression profile of *CCD4* (LOC103321735) in ‘ZLE’ rapidly rose and achieved 22.5 times higher than that of ‘HJH’ at S3. This implies that *CCD4* might be bound up with the flower coloration differences of ‘HJH’ and ‘ZLE’. Previous studies have shown that CCDs could degrade carotenoids in *Chrysanthemum* [28], *Rhododendron japonicum* [56], *L. brownie* [57], *P. persica* [32], *O. fragrans* [58] and *Arabidopsis* [59]. We speculated that the *CCD4* gene had low expression, leading to carotenoid accumulation in ‘HJH’. At the same time, *CCD4* was highly expressed in ‘ZLE’, resulting in flower color differences between the two varieties.

### 3.3. Potential Regulatory Genes Related to Carotenoids in P. mume

Comprehensive analysis of metabolome and transcriptome has increasingly become a popular and practical tool for exploring new genes that may participate in various metabolic pathways [60]. This study combined metabolome and transcriptome data to clarify the correlation between genes and carotenoids in *P. mume*. This provides valuable information for studying the accumulation and regulation of carotenoids. Five genes that were significantly related to lutein and β-carotene were screened through correlation network analysis (*PSY*, *CRTISO*, *CHYE*, *ZEP* and *CCD4*). Mainly, *CCD4* was both correlated with lutein and β-carotene. Furthermore, a significant negative correlation between *CCD4* gene and metabolites was found. We guessed that *CCD4* might play a key role in carotenoid accumulation.

Carotenoid biosynthesis is strictly regulated in plants, and transcription factors play an important role. Previous reports showed that *CsMADS5*, *CsMADS6* and *CsERF06*1 in citrus [61,62,63], *UpMYB44* in *Ulva prolifera* [64], *MdAP2-34* in *M. domestica* [65] and *SlNAC1*, *SlNAC4*, *SlAP2a*, and *SlBBX20* in *Solanum lycopersicum* [66,67,68,69] could directly interact with the carotenoid synthesis gene to regulate carotenoid accumulation. In *P. mume*, the transcriptional regulation mechanism of carotenoids is unclear, hindering the germplasm innovation of flower color. To explore the potential regulatory network of flower color differences, co-expression analysis was carried out between carotenoid pathway genes and differentially expressed transcription factors (TFs). There were 93 TFs of 19 TF families that had a significant correlation with five genes. And bHLH family (21 unigenes) was the most abundant. bHLH families, which belong to superfamily transcription factors, are widely spread in plants [70]. In *Carica papaya*, *CpbHLH1* and *CpbHLH2* combine with downstream structural genes *CpCYCB* and *CpLCYB* to activate or transcribe, respectively [71]. Among the 93 TFs, 33 were involved in the regulation of *CCD4*. *bHLH63*, *ASIL1*, *bHLH148*, *MYB114*, *RAP2-7* and *ERF3* were positively correlated with *CCD4*. we conjectured that the six TFs probably regulated *CCD4* to affect the accumulation of lutein and β-carotene. Especially, the *bHLH63*, which belongs to the bHLH family, had the strongest connection with *CCD4*. Notably, *AtCIB1* (AT4G34530) and *AtTOE1* (AT2G28550) were the *bHLH63* and *RAP2-7* homologous protein in *Arabidopsis*. *AtCIB1* interacts with *AtCRY2* (cryptochrome 2) to positively regulate flower development in *Arabidopsis* [72]. *AtTOE1* was involved in the timing of flowering in *Arabidopsis* [73]. We considered it would be a significant study to determine the relationship between these transcription factors and carotenoids through *PmCCD4*. Meanwhile, it also provides the scientific basis for the innovation of flower color breeding in *Prunus*.

### 3.4. PmCCD4 Reduced Carotenoids in Transgenic Tobacco

CCDs can cause oxidative cleavage carotenoid molecules at one or both ends, which is one of the main pathways for carotenoid cleavage in plants. Recently, more studies have shown that *CCD1* and *CCD4* are critical genes involved in carotenoid metabolism, which impacts plants’ color and aroma formation [74,75]. Among them, CCD4 is located in the plastids and can directly interact with carotenoids to perform catalytic functions. CCDs require four conserved histidine residues to bind divalent iron ions as cofactors to exert their catalytic activity [76]. Bioinformatics analysis revealed that PmCCD4 had typical conserved domains, stable β-propeller and α-helical secondary structures, and four conserved histidine residues, ensuring its enzymatic activity. Phylogenetic analysis indicated that PmCCD4 was closely related to PpCCD4, MdCCD4 and RdCCD4, with a homology of 98.16% to PpCCD4. Previous research demonstrated that PpCCD4, MdCCD4 and RdCCD4 had the ability to cleave carotenoids in vivo or in vitro, producing volatile compounds or other apocarotenoids [77,78]. Consequently, we proposed that *PmCCD4* might also cleave carotenoids, leading to color differences in the ‘HJH’ and ‘ZLE’ petals. We identified the function of *PmCCD4* through heterologous overexpression in tobacco. The results showed that transgenic lines with *PmCCD4* had significantly lower content of lutein, β-carotene, violaxanthin and neoxanthin than the WT, particularly lutein, which decreased by 5.8-fold. This result indicates that PmCCD4 could cleave carotenoids in tobacco. And lutein might be the primary substrate for PmCCD4. However, whether the substrate of PmCCD4 is lutein and whether there are other carotenoid substrates still need further exploration. The next investigations can be carried out using vitro enzyme activity assays by adding specific substrates to provide additional evidence. In addition, the cleavage of carotenoids by the *CCD4* gene can generate volatile compounds. We speculated that *PmCCD4* might be involved in the formation of floral or fruity scents in *P. mume*. This will be a highly intriguing topic. These findings will facilitate the breeding of flower color in *P. mume*. Meanwhile, they also provide the scientific basis for the innovation of flower color breeding in *Prunus*.

## 4. Material and Methods

### 4.1. Plant Materials

The *P. mume* ‘Huangjinhe’ (‘HJH’) and ‘Zaolve’ (‘ZLE’) were planted in Chong qing, China (105° 40’ E, 29° 39’ N) and grown in a natural environment. The five opening stages of flowers (small bud stage (S1), big bud stage (S2), half-flowering stage (S3), full-blooming stage (S4), terminal florescence (S5)) were sampled from the two varieties in February 2021. The above samples were stored at −80 °C for standby. There were three biological replicates for each experiment.

### 4.2. Petal Color Measurement

Based on the Royal Horticultural Society Color Chart (RHSCC, British, 2007), the colors of five opening stages of petals were defined. Spectrophotometer NF555 (Nippon Denshoku Industries Co. Ltd., Tokyo, Japan) measured petal color parameters with CIE system C/20° illumination conditions. The light-collecting port was aligned in the middle of the petal for measurement. Each stage was measured 20 times and finally taken as the average value. Chroma (C) and hue angle (h) were calculated through the following equations: C = (a*^2^ + b*^2^)^1/2^ and h = arctan(b*/a*) [79].

### 4.3. Metabolome Analysis

The flavone metabolome and carotenoid metabolome were extracted from frozen flowers by the manuals of MetWare (Wuhan, China). The contents of flavonoids and carotenoids were detected based on the AB Sciex QTRAP 6500 LC-MS/MS platform. Scheduled multiple-reaction monitoring (MRM) was applied to analyze flavonoids and carotenoids. Analyst 1.6.3 software (Sciex) and Multiquant 3.0.3 software (Sciex) were used to quantify all metabolites.

Based on the orthogonal partial-least-squares discriminant analysis (OPLS-DA) model, the differential metabolites (‘ZLE’-S2 vs. ‘HJH’-S2, ‘ZLE’-S3 vs. ‘HJH’-S3 and ‘ZLE’-S4 vs. ‘HJH’-S4) were identified when a project (VIP) value ≥1 and|log2 Fold Change (FC)| ≥ 1.

### 4.4. RNA Isolation, Library Establishment, and Sequencing

Total RNA was isolated from three stages (S2, S3 and S4) in two varieties with three biological replicates. After RNA quality was assessed to be qualified, mRNA was enriched with PolyA tail by oligo (DT) magnetic beads. The first strand of cDNA was synthesized using short RNA fragments broken with a fragmentation buffer as templates. Then, the double-stranded cDNA was synthesized through buffer, dNTPs and DNA polymerase. Next, double-stranded cDNA purification, terminal repair, tail additions and adaptor ligation were carried out. Afterwards, the final cDNA library was obtained by selecting fragment size and PCR enrichment. The library passed the quality inspection and sequenced with Illumina Hiseq 4000 platform (Metware, Wuhan, China).

### 4.5. Transcriptome Assembly and Gene Annotation

By removing adapter reads, low-quality paired reads and equivocal paired reads, the raw data were filtered. Data quality was checked with sequencing error rate distribution (Q20 and Q30) and GC content distribution. The high-quality clean reads were applied to assembly and subsequent analysis. Sequence alignment of clean reads with the reference genome (https://www.ncbi.nlm.nih.gov/genome/?term=prunus+mume+, accessed on 13 September 2021) was conducted using HISAT2.

The raw data were conserved in the National Center for Biotechnology Information (NCBI) database (accession no. PRJNA854285). Assembly sequences were compared and annotated with NCBI nonredundant protein sequences (NR), Kyoto Encyclopedia of Genes and Genomes (KEGG), Swiss-Prot, Translation from EMBL database (TrEMBL), Clusters of Orthologous Groups of protein (KOG), Gene Ontology (GO), and Protein Families (Pfam). Principal component analysis (PCA) was used to verify sample variability.

### 4.6. Differentially Expressed Genes Screening

The gene-expression levels were calculated through fragments per kilobase of transcription per million fragments mapped (FPKM). The gene differential expression was analyzed in term of DESeq2 v.1.22.2 software in R package. Differentially expressed genes (DEGs), in which ‘ZLE’-S2 vs. ‘HJH’-S2, ‘ZLE’-S3 vs. ‘HJH’-S3 and ‘ZLE’-S4 vs. ‘HJH’-S4 were found, were identified on the basis of |log2 Fold Change (FC)| ≥ 1 and false discovery rate (FDR) < 0.05.

### 4.7. Quantitative Real-Time PCR Analysis of Screened Gene

A totals of 12 DEGs in the carotenoid biosynthesis pathway were analyzed using qRT-PCR. Screened genes and specific primers were displayed in Supplementary Table S7. The protein phosphatase 2A (*PP2A*) gene of *P. mume* was considered as an internal control. First-strand cDNA synthesis was reversed using PrimeScriptTMRT Reagent Kit with gDNA Eraser (Takara, Dalian, China). The template used cDNA (2 µL) in a 10 µL qRT-PCR by TB Green II Premix Ex Taq (Takara, Dalian, China) under the following conditions: 95 °C for 30 s and 40 cycles of 95 °C for 5 s, and 60 °C for 30 s. The 2^−ΔΔCt^ method was applied to calculate relative expression levels, and at least three biological replicates were performed.

### 4.8. Gene Clone and Protein Analysis

Primers were designed to clone the *PmCCD4* sequence using ‘HJH’ blooming-stage petal cDNA as a template (Supplementary Table S11). A phylogenetic tree was constructed to determine the homology by employing MEGA7.0 with the neighbor-joining (NJ) method [80]. A total of 1000 iterations of bootstrap tests were executed to support the calculation relationship of the phylogenetic tree, which was later visualized through iTOL (https://itol.embl.de/, accessed on 13 March 2023) [81]. DNAMAN was utilized to align the CCD amino acid sequence. SWISS-MODEL (https://swissmodel.expasy.org/, accessed on 20 March 2023) and Pymol software were employed to build the PmCCD protein model and analyze its secondary structure [82].

### 4.9. Subcellular Localization Analysis

Subcellular localization was predicted using PCLR (http://www.andrewschein.com/cgi-bin/pclr/pclr.cgi, accessed on 20 March 2023), and specific primers (*GFP-PmCCD4-F*/*GFP-Pm-CCD4-R*) were designed to construct the pCAMBIA1300-*PmCCD4*-GFP vector (Supplementary Table S11). Recombinant plasmids and the control plasmid PT-RK were simultaneously transformed into blossom protoplasts of *P. mume*. The method was based on the Bao protocol [83], with slight modifications. Protoplasts were cultured for 24 h in the dark at 22 °C, followed by observation and photography under a laser confocal microscope Leica TCS SP8.

### 4.10. Determination of Carotenoids from PmCCD4-Overexpressing Transgenic Tobacco

Specific primers (*sp1300-PmCCD4-F*/*sp1300-PmCCD4-R*) were designed to construct the pCAMBIA1300-*PmCCD4* vector for transformation into GV3101 agrobacterium (Supplementary Table S11). Then, using the leaf disc transformation method as previously described, the vector was introduced into tobacco NC89 [84]. Total RNA was isolated from stems, leaves, flowers and fruits of *PmCCD4*-overexpressing transgenic tobacco. First-strand cDNA synthesis was reversed for qRT-PCR. The *NtActin* gene of tobacco was considered an internal control (Supplementary Table S7). The total carotenoid content was determined with slight modifications to a previous report [47]. Leaves were grinded into a fine powder using liquid nitrogen and divided into 0.1 g. Next, 5 mL of methanol solution was added, and it was shaken to mix. The mixture was extracted for 24 h in the dark at 4 °C. The supernatants collected by centrifugation (12,000× rpm, 10 min) were measured the absorbance at 649, 665 and 470 nm. The calculation of total carotenoids was based on the following formula: Chlorophyll a (μg/g) = 16.29A665 − 8.54A649 × 10, chlorophyll b (μg/g) = 30.66A649 − 13.58A665 × 10; total chlorophylls (μg/g) = 2.71A665 + 22.12A649 × 10; and total carotenoids (μg/g) = (1000A470 − 1.63Chla − 104.96Chlb) ÷ 221 × 10. The method for extracting and analyzing carotenoids in tobacco leaves using HPLC referred to the manuals of *P. mume* carotenoids.

### 4.11. Statistical Analyses

Microsoft Excel 2020 and SPSS Statistics 24.0 software (IBM SPSS, Chicago, IL, USA) conducted the data analysis. The heatmaps were presented with TBtools v2.001 software [85]. The co-expression network was performed using Metware Cloud (https://cloud.metware.cn/#/home, accessed on 22 March 2023) and visualized by Cytoscape v 3.9.1 [86].

## 5. Conclusions

In our study, a combined transcriptome and metabolome of three flowering stages in ‘HJH’ and ‘ZLE’ were analyzed to explore the coloration mechanism of yellow flowers in *P. mume*. We determined that carotenoids, rather than flavonoids, made contributions to yellow flowers. And lutein was the key pigment for the yellow color in *P. mume*. Meanwhile, we identified six major transcription factors that likely regulated *PmCCD4* to affect the accumulation of lutein. Furthermore, it had been preliminarily determined that lutein might be the primary substrate for *PmCCD4* in transgenic tobacco by HPLC. We speculated that *PmCCD4* might be involved in the cleavage of lutein in plastids, thereby affecting the formation of yellow flowers in *P. mume*. These results could establish a material basis for improving flower color and enriching the candidate gene pool in *P. mume*. Furthermore, to investigate the substrate affinity of PmCCD4, in vitro enzyme activity assays can be conducted by adding specific substrates. In addition, whether *PmCCD4* is involved in the aroma formation of flowers and fruits in *P. mume* deserves further research.

## Figures and Tables

**Table 1:** Chrominance information of *P. mume* ‘HJH’ and ‘ZLE’ flowers (*n* = 3).

Samples and Stages	RHSCC	L*	a*	b*	C*	*h*
HJH-S1	5D	82.45	−6.97	32.69	33.43	−77.97
HJH-S2	5D	83.84	−6.72	33.49	34.16	−78.65
HJH-S3	2D	89.97	−6.53	26.42	27.22	−76.12
HJH-S4	155D	94.75	−5.78	15.50	16.55	−69.55
HJH-S5	155B	92.76	−7.17	20.28	21.51	−70.52
ZLE-S1	4D	91.95	−8.33	23.57	25.00	−70.53
ZLE-S2	155A	93.88	−7.35	17.10	18.61	−66.73
ZLE-S3	155D	95.82	−6.97	12.44	14.26	−60.75
ZLE-S4	155D	95.95	−6.85	12.47	14.23	−61.20
ZLE-S5	155D	95.30	−6.47	12.95	14.47	−63.46

Note: RHSCC means Royal Horticultural Society Color Chart. The a* value represents the red–green axis, with positive values indicating red and negative values indicating green. The b* value represents the yellow–blue axis, with positive values indicating yellow and negative values indicating blue. L* value denotes lightness, C* value denotes chroma and *h* value denotes hue angle.

## Data Availability

The original contributions presented in the study are included in the article/Supplementary Materials; further inquiries can be directed to the corresponding authors.

## Supplementary Materials

The following supporting information can be downloaded at: https://www.mdpi.com/article/10.3390/plants12183333/s1, Figure S1: Transcriptome profiles analysis of *P. mume* ‘HJH’ and ‘ZLE’ at S2, S3 and S4. (A) The correlation index R2 of biological repetition. Numbers represent sample correlation. (B) Go classification of DEGs. The *x*-axis denotes secondary go items, and the *y*-axis denotes the number of DEGs of go items. Right blocks mean GO classification. Figure S2: MA-plot and volcano plots for DEGs of 3 comparison groups in *P. mume* ‘HJH’ and ‘ZLE’ at S2, S3 and S4. (A) MA-plot of ‘ZLE’-S2 vs. ‘HJH’-S2. *X*-axis means log2FC value and *y*-axis means the average value of gene expression in the two samples. (B) volcano plot of ‘ZLE’-S2 vs. ‘HJH’-S2. The *x*-axis shows the change of gene-expression multiples, and the *y*-axis shows the significance level of DEGs. (C) MA-plot of ‘ZLE’-S3 vs. ‘HJH’-S3. (D) volcano plot of ‘ZLE’-S3 vs. ‘HJH’-S3. (E) MA-plot of ‘ZLE’-S4 vs. ‘HJH’-S4. (F) volcano plot of ‘ZLE’-S4 vs. ‘HJH’-S4. Red, green and blue represent up-regulated, down-regulated and non-DEGs, respectively. Figure S3: Top 20 KEGG pathways enrichment analysis of 3 comparison groups *P. mume* ‘HJH’ and ‘ZLE’ at S2, S3 and S4. (A) ‘ZLE’-S2 vs. ‘HJH’-S2. (B) ‘ZLE’-S3 vs. ‘HJH’-S3. (C) ‘ZLE’-S4 vs. ‘HJH’-S4. Figure S4: Multiple sequence alignment between PmCCD4 and other CCD4s. Red box indicates histidine residue. Green dot indicates glutamate residue. Blue dot indicates aspartate residue. Table S1: Information of 74 flavonoids compounds between ‘HJH’ and ‘ZLE’ flower at S2, S3 and S4 in *P. mume*. Table S2: Information of 20 differential flavonoids metabolites between ‘HJH’ and ‘ZLE’ flower at S2, S3 and S4 in *P. mume*. Table S3: Information of 37 carotenoids compounds between ‘HJH’ and ‘ZLE’ flower at S2, S3 and S4 in *P. mume*. Table S4: Information of 28 differential carotenoid metabolites between ‘HJH’ and ‘ZLE’ flower at S2, S3 and S4 in *P. mume*. Table S5: Overview of the transcriptome sequencing dataset and quality check. Table S6: Summary of functional annotation for assembled genes. Table S7: Primers used in the qRT-PCR. Table S8: Correlation of carotenoid pathway genes with 5 carotenoid metabolites. Table S9: Information of 93 TFs in the network. Table S10: Correlation of 93 TFs with 5 screened genes. Table S11: Primers used to construct vector.
