# Supplementary material for: Integrative Analysis of Metabolome and Transcriptome Revealed Lutein Metabolism Contributed to Yellow Flower Formation in Prunus mume
Source: Plants (Basel). 2023 Sep 21;12(18):3333. doi: 10.3390/plants12183333 (PMC10537319; doi:10.3390/plants12183333)
Supplement: Supplementary file 1 [file plants-12-03333-s001.zip › plants-2597310 -Supplementary.pdf]

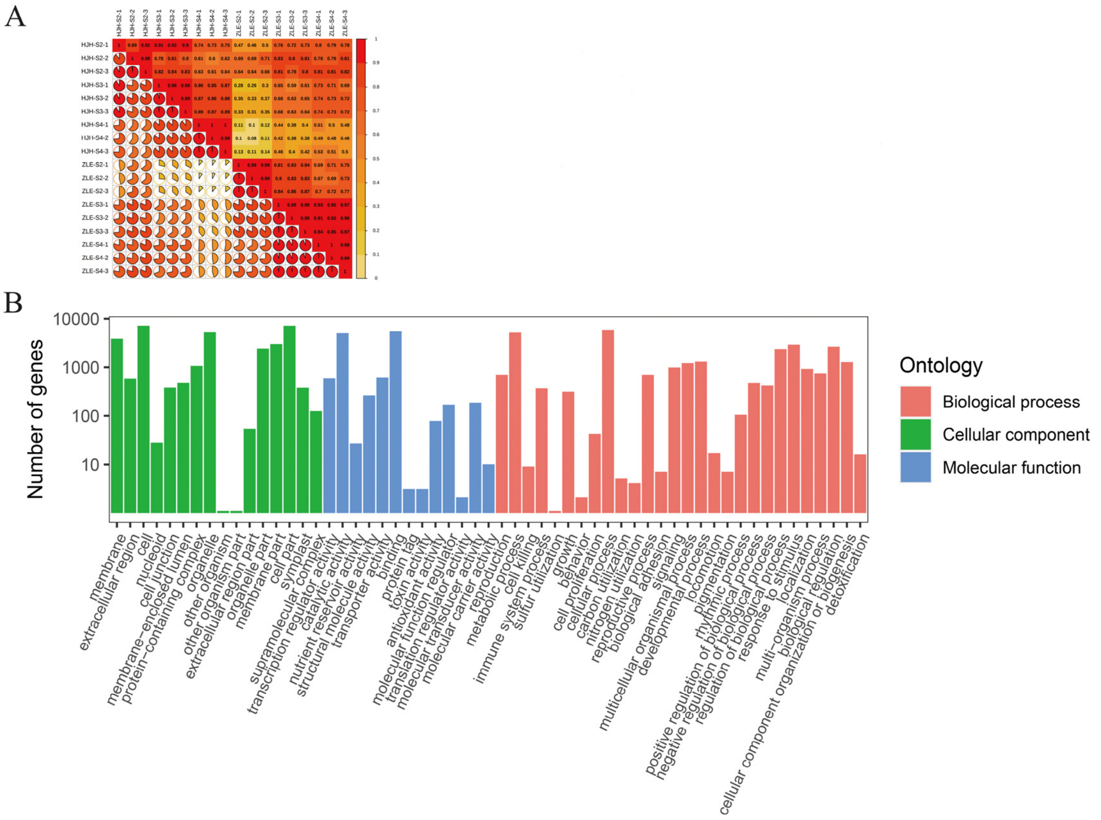

Supplementary Figure S1

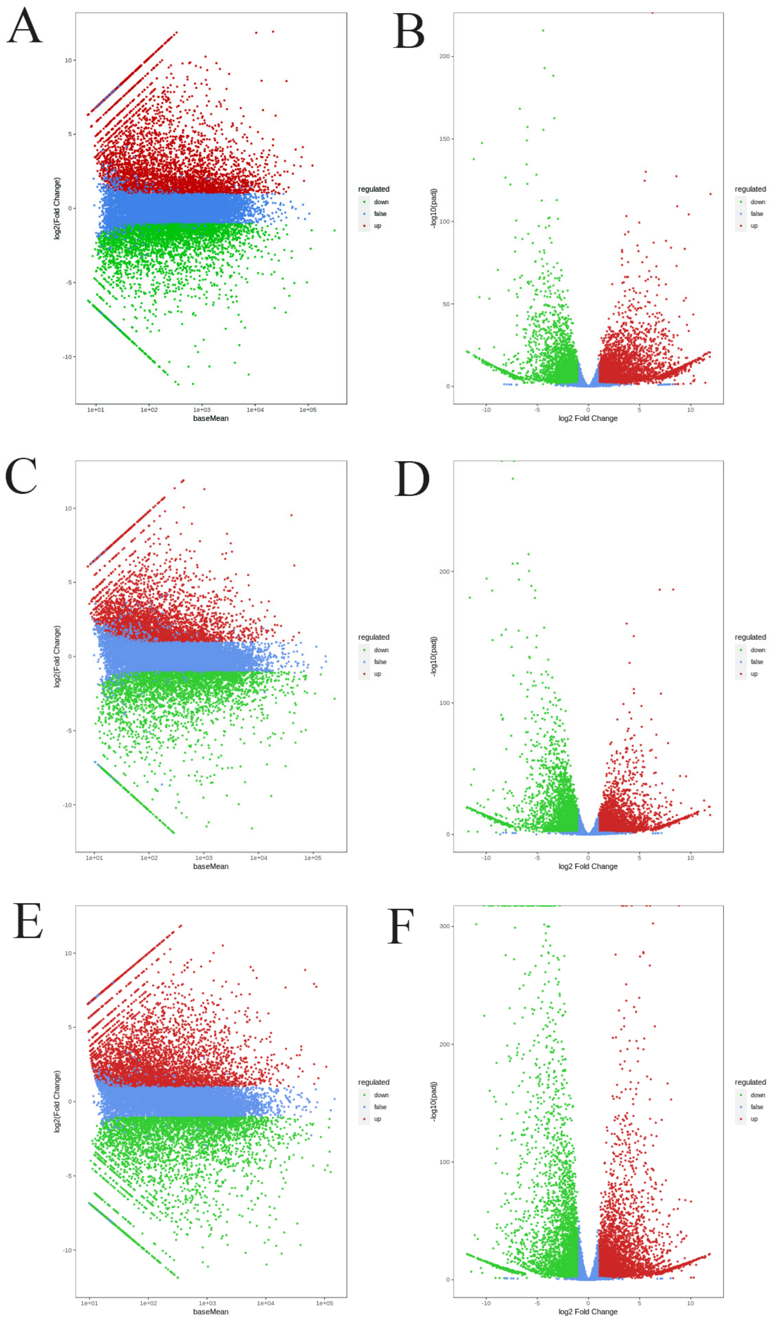

Supplementary Figure S2

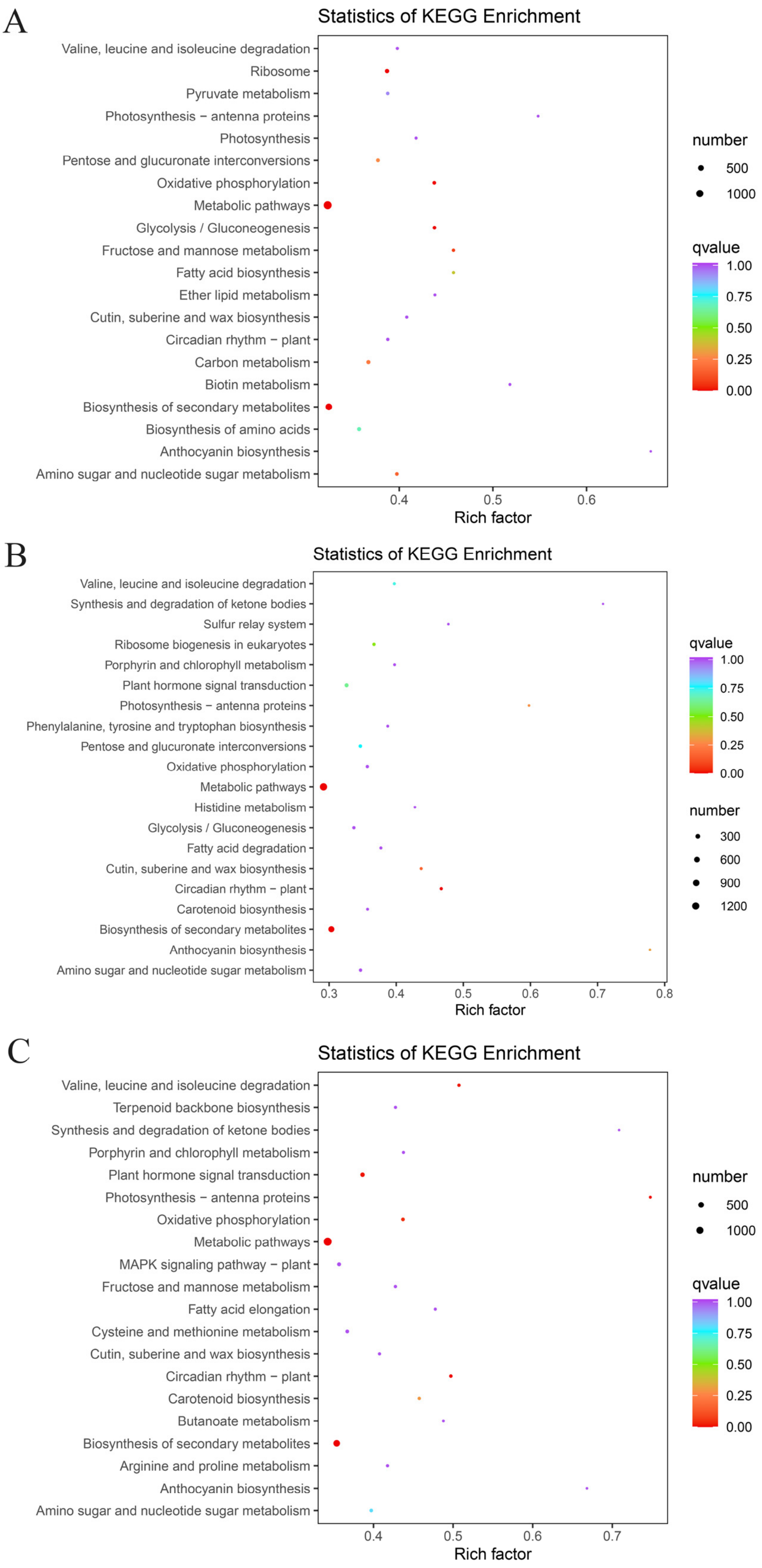

Supplementary Figure S3

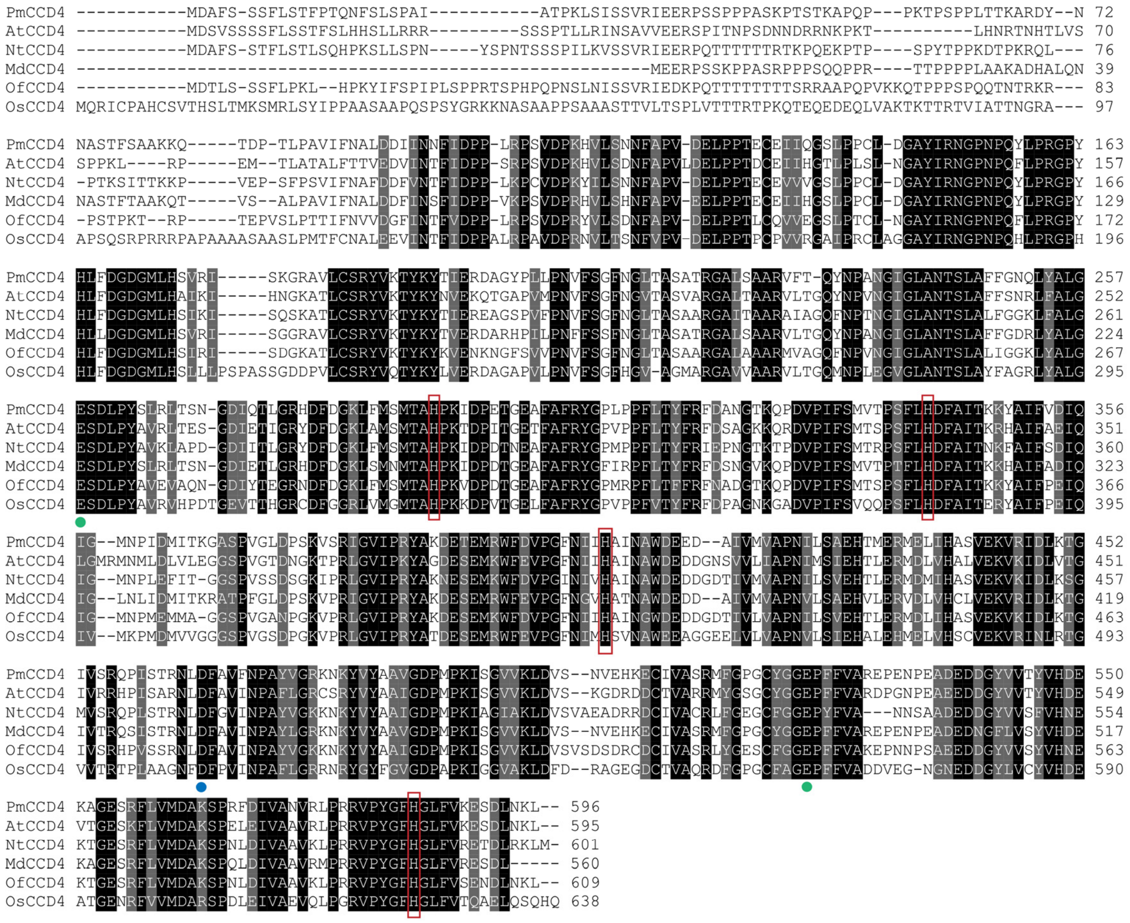

Supplementary Figure S4
